# 2-Methyl-1,1-diphenyl-2-[(4*S*)-4-phenyl-4,5-di­hydro-1,3-oxazol-2-yl]propan-1-ol

**DOI:** 10.1107/S1600536813014670

**Published:** 2013-06-08

**Authors:** Wen-Xiao Jia, Yu-Lai Hu, Dang-Feng Huang, Teng Niu, Yan-Jun Ma

**Affiliations:** a College of Chemistry and Chemical Engineering, Northwest Normal University, Lanzhou, Gansu Province 730070, People’s Republic of China

## Abstract

In the title compound, C_25_H_25_NO_2_, the phenyl ring on the 1,3-oxazole ring is disordered over two positions with occupancies of 0.600 (4) and 0.400 (4). The inter­planar angle between these two disordered rings is 77.8 (2)°. There is an intra­molecular O—H⋯N hydrogen bond of moderate strength. In the crystal, C—H⋯π inter­actions interconnect neighbouring molecules. The absolute structure has been derived from the known absolute structure of the reagents.

## Related literature

For the synthesis and applications of oxazolines, see: Ghosh *et al.* (1998 ▶); Johnson & Evans (2000 ▶). For the categorization of hydrogen bonds, see: Gilli & Gilli (2009 ▶).
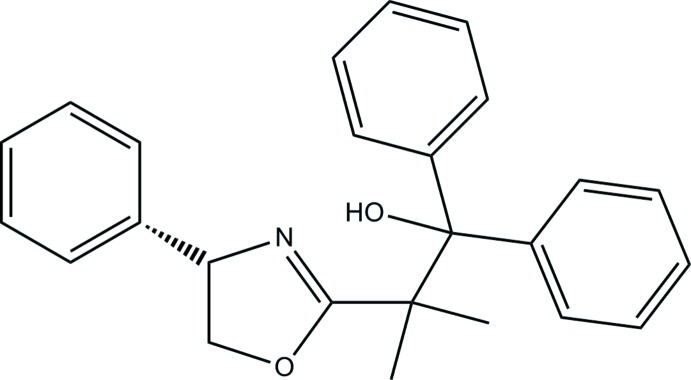


## Experimental

### 

#### Crystal data

C_25_H_25_NO_2_
*M* *_r_* = 371.46
Orthorhombic, 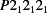

*a* = 9.5405 (2) Å
*b* = 10.9430 (9) Å
*c* = 19.2901 (6) Å
*V* = 2013.92 (18) Å^3^
*Z* = 4
Mo *K*α radiation
μ = 0.08 mm^−1^
*T* = 293 K
0.34 × 0.07 × 0.06 mm

#### Data collection

Bruker APEXII CCD diffractometer
Absorption correction: multi-scan (*SADABS*; Bruker, 2008 ▶) *T*_min_ = 0.849, *T*_max_ = 0.977
5590 measured reflections
2428 independent reflections
2103 reflections with *I* > 2σ(*I*)
*R*_int_ = 0.040

#### Refinement

*R*[*F* ^2^ > 2σ(*F* ^2^)] = 0.045
*wR*(*F* ^2^) = 0.102
*S* = 1.08
2428 reflections
305 parameters
338 restraints
H atoms treated by a mixture of independent and constrained refinement
Δρ_max_ = 0.21 e Å^−3^
Δρ_min_ = −0.17 e Å^−3^

### 

Data collection: *APEX2* (Bruker, 2008 ▶); cell refinement: *SAINT* (Bruker, 2008 ▶); data reduction: *SAINT*; program(s) used to solve structure: *SHELXS97* (Sheldrick, 2008 ▶); program(s) used to refine structure: *SHELXL97* (Sheldrick, 2008 ▶); molecular graphics: *SHELXTL* (Sheldrick, 2008 ▶) and *DIAMOND* (Brandenburg, 2010 ▶); software used to prepare material for publication: *SHELXTL* and *publCIF* (Westrip, 2010 ▶).

## Supplementary Material

Crystal structure: contains datablock(s) I, global. DOI: 10.1107/S1600536813014670/fb2285sup1.cif

Structure factors: contains datablock(s) I. DOI: 10.1107/S1600536813014670/fb2285Isup2.hkl

Supplementary material file. DOI: 10.1107/S1600536813014670/fb2285Isup3.cml

Additional supplementary materials:  crystallographic information; 3D view; checkCIF report

## Figures and Tables

**Table 1:** Hydrogen-bond geometry (Å, °) *Cg*2, *Cg*3 and *Cg*4 are the centroids of the C8–C13, C14–C19 and C20–C25 rings, respectively.

*D*—H⋯*A*	*D*—H	H⋯*A*	*D*⋯*A*	*D*—H⋯*A*
O2—H2⋯N6	0.86 (3)	1.89 (3)	2.712 (3)	159 (3)
C1—H1⋯*Cg*2^i^	0.98	2.80	3.771 (3)	173
C2—H2*A*⋯*Cg*2^ii^	0.97	2.86	3.589 (3)	132
C24*B*—H24*B*⋯*Cg*3^iii^	0.93	2.87	3.776 (6)	166
C24*B*—H24*B*⋯*Cg*4^iii^	0.93	2.97	3.785 (9)	147

Symmetry codes: (i) 
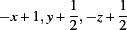
; (ii) 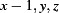; (iii) 
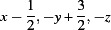
.

**Fig. 1.**
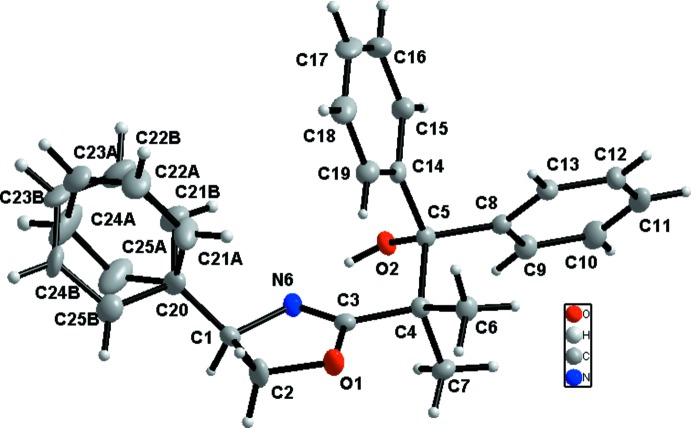
The title molecule with the atomic labelling scheme. The displacement ellipsoids are shown at the 50% probability level.

**Fig. 2.**
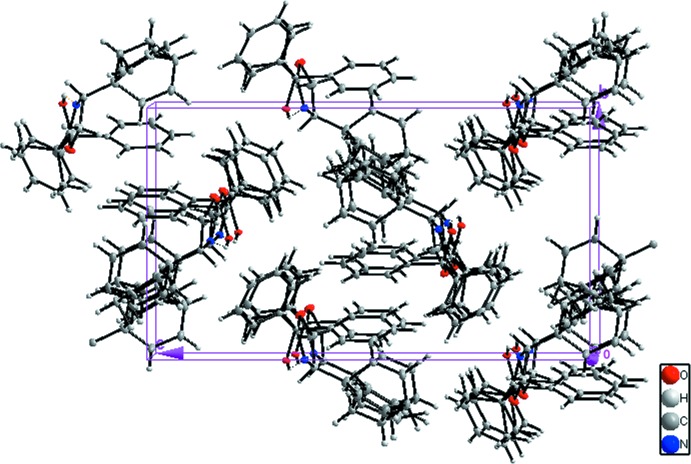
Packing diagram of the title molecules viewed approximately along the a axis.

## supplementary crystallographic information

### Comment

Over the last decade, C2-symmetric chiral oxazoline metal complexes have been recognized as an effective class of chiral catalyst in a variety of transition metal catalyzed asymmetric reactions. Thus, the design and synthesis of new chiral oxazoline ligands has inspired many scientists to work with great efforts (Ghosh *et al.*, 1998, Johnson & Evans, 2000).

The title compound (Fig. 1) is a new oxazoline ligand, (*S*)-2-methyl-1,1-diphenyl-2-(4-phenyl-4,5-dihydrooxazol-2-yl propan-1-ol), which has been designed as a potential ligand for asymmetric catalysis. It combines diphenyl methyl units and chiral oxazoline ring together with dimethyl methyl malonate.

Fig. 2 shows the packing of the molecules in the title structure.

Peculiarity of the title crystal structure is presence of a disorder which affects one of the phenyl rings which is split into two positions: C20//C21*a*//C22*a*//C23*a*//C24*a*//C25*a* and C20//C21*b*//C22*b*//C23*b*//C24*b*//C25*b*, the respective occupations of which are 0.600 (4) and 0.400 (4). The interplanar angle of both disordered rings equals to 77.8 (2)°.

There is an intramolecular hydrogen bond O2—H2···N6 of moderate strength in the structure (Tab. 1). (For categorization of the hydrogen bonds, see Gilli & Gilli, 2009.) There are present C—H···π-electron ring interactions in the structure (Tab. 1), too.

### Experimental

Mono(oxazoline) (0.5 g, 2 mmol) dissolved in 10 ml of tetrahydrofuran was placed into a two-neck 50 ml round-bottom flask that had been previously dried and that was equipped with a magnetic stirrer and a nitrogen inlet. The solution was cooled to 273 K and phenylmagnesium bromide (1.4 g, 8 mmol) which had been dissolved in 2 ml of tetrahydrofuran was added dropwise under nitrogen atmosphere to the solution of monoxazoline in tetrahydrofuran. The reaction mixture was stirred for 2 h at the same temperature. Then thin layer chromatography showed that the raw material had disappeared and indicated completion of the reaction. In the following step, 15 ml of saturated NH_4_Cl was added into the reaction solution at 273 K. The product was extracted by ethyl acetate (3×10 ml). The combined ethyl acetate extracts were dried over Na_2_SO_4_. The residue obtained after the evaporation of the solvent was purified by silica gel column chromatography with petroleum ether. The crude product was dissolved in 1 ml petroleum ether and the crystals were recrystallized after 4 hours in 55% yield as white or colourless needles typically 2-3 mm long and 0.5 mm wide. Melting point: 380-381 K (determined by a X-4 digital display microscopic melting-point apparatus).

### Refinement

The H atoms which have not been involved in the disordered phenyl rings were discernible in the difference electron density maps. The H atoms which were attached to the carbons were situated into the idealized positions and constrained using the following constraints: C_aryl_—H_aryl_ = 0.93; C_methyl_—H_methyl_ = 0.96; C_methylene_—H_methylene_ = 0.97; C_methine_—H_methine_ = 0.98 Å. *U*_iso_H_aryl_ = 1.2*U*_eq_C_aryl_; *U*_iso_H_methyl_ = 1.5*U*_eq_C_methyl_; *U*_iso_H_methylene_ = 1.2*U*_eq_C_methylene_; *U*_iso_H_methine_ = 1.2*U*_eq_C_methine_. The positional parameters of the hydroxyl hydrogen were freely refined while *U*_iso_H_hydroxyl_ = 1.5*U*_eq_O_hydroxyl_. The occupational parameters of the disoredered phenyl rings were constrained in such a way that the their sum equalled to 1. The displacement parameters of the corresponding disordered atoms C21*a*, C21*b* ··· C25*a*, C25*b* were restrained by the command ISOR 0.01 0.02 and SIMU_* (*SHELXL*97, Sheldrick, 2008). In absence of significant resonant scatterers 1284 Friedel pairs have been merged by application of the command MERG 3 [*SHELXL*97 (Sheldrick, 2008)].

### Crystal data

**Table:**

C_25_H_25_NO_2_	*D*_x_ = 1.225 Mg m^−^^3^
*M**_r_* = 371.46	Melting point = 380–381 K
Orthorhombic, *P*2_1_2_1_2_1_	Mo *K*α radiation, λ = 0.71073 Å
Hall symbol: P 2ac 2ab	Cell parameters from 2137 reflections
*a* = 9.5405 (2) Å	θ = 3.0–28.6°
*b* = 10.9430 (9) Å	µ = 0.08 mm^−^^1^
*c* = 19.2901 (6) Å	*T* = 293 K
*V* = 2013.92 (18) Å^3^	Block, colourless
*Z* = 4	0.34 × 0.07 × 0.06 mm
*F*(000) = 792	

### Data collection

**Table:**

Bruker APEXII CCD diffractometer	2428 independent reflections
Radiation source: fine-focus sealed tube	2103 reflections with *I* > 2σ(*I*)
Graphite monochromator	*R*_int_ = 0.040
ω scans	θ_max_ = 26.8°, θ_min_ = 3.0°
Absorption correction: multi-scan (*SADABS*; Bruker, 2008)	*h* = −12→4
*T*_min_ = 0.849, *T*_max_ = 0.977	*k* = −10→13
5590 measured reflections	*l* = −24→21

### Refinement

**Table:**

Refinement on *F*^2^	Secondary atom site location: difference Fourier map
Least-squares matrix: full	Hydrogen site location: inferred from neighbouring sites
*R*[*F*^2^ > 2σ(*F*^2^)] = 0.045	H atoms treated by a mixture of independent and constrained refinement
*wR*(*F*^2^) = 0.102	*w* = 1/[σ^2^(*F*_o_^2^) + (0.0298*P*)^2^ + 0.5879*P*] where *P* = (*F*_o_^2^ + 2*F*_c_^2^)/3
*S* = 1.08	(Δ/σ)_max_ < 0.001
2428 reflections	Δρ_max_ = 0.21 e Å^−^^3^
305 parameters	Δρ_min_ = −0.17 e Å^−^^3^
338 restraints	Extinction correction: *SHELXL97* (Sheldrick, 2008), Fc^*^=kFc[1+0.001xFc^2^λ^3^/sin(2θ)]^-1/4^
114 constraints	Extinction coefficient: 0.0119 (14)
Primary atom site location: structure-invariant direct methods	

### Special details

**Table:**

Geometry. All e.s.d.'s (except the e.s.d. in the dihedral angle between two l.s. planes) are estimated using the full covariance matrix. The cell e.s.d.'s are taken into account individually in the estimation of e.s.d.'s in distances, angles and torsion angles; correlations between e.s.d.'s in cell parameters are only used when they are defined by crystal symmetry. An approximate (isotropic) treatment of cell e.s.d.'s is used for estimating e.s.d.'s involving l.s. planes.
Refinement. Refinement of *F*^2^ against ALL reflections. The weighted *R*-factor *wR* and goodness of fit *S* are based on *F*^2^, conventional *R*-factors *R* are based on *F*, with *F* set to zero for negative *F*^2^. The threshold expression of *F*^2^ > σ(*F*^2^) is used only for calculating *R*-factors(gt) *etc*. and is not relevant to the choice of reflections for refinement. *R*-factors based on *F*^2^ are statistically about twice as large as those based on *F*, and *R*- factors based on ALL data will be even larger.

### Fractional atomic coordinates and isotropic or equivalent isotropic displacement parameters (Å^2^)

**Table:**

	*x*	*y*	*z*	*U*_iso_*/*U*_eq_	Occ. (<1)
O1	0.28330 (17)	0.33991 (18)	0.16867 (10)	0.0264 (5)	
O2	0.68088 (19)	0.51237 (16)	0.19226 (9)	0.0183 (4)	
H2	0.596 (3)	0.533 (3)	0.1828 (15)	0.027*	
C14	0.6840 (3)	0.3818 (2)	0.09158 (12)	0.0183 (5)	
C3	0.4060 (3)	0.4020 (2)	0.17253 (12)	0.0176 (5)	
C15	0.8082 (3)	0.4282 (3)	0.06375 (13)	0.0256 (6)	
H15	0.8770	0.4579	0.0935	0.031*	
N6	0.4060 (2)	0.5148 (2)	0.15641 (10)	0.0196 (5)	
C13	0.8502 (3)	0.2144 (2)	0.17551 (13)	0.0214 (6)	
H13	0.8097	0.1824	0.1356	0.026*	
C8	0.7957 (2)	0.3207 (2)	0.20445 (12)	0.0171 (5)	
C11	1.0271 (3)	0.2014 (3)	0.26401 (14)	0.0266 (6)	
H11	1.1042	0.1623	0.2835	0.032*	
C12	0.9640 (3)	0.1553 (3)	0.20530 (13)	0.0239 (6)	
H12	0.9980	0.0838	0.1854	0.029*	
C10	0.9738 (3)	0.3071 (3)	0.29357 (14)	0.0269 (6)	
H10	1.0151	0.3389	0.3334	0.032*	
C19	0.5830 (3)	0.3393 (3)	0.04617 (13)	0.0231 (6)	
H19	0.4987	0.3090	0.0632	0.028*	
C16	0.8312 (3)	0.4309 (3)	−0.00695 (14)	0.0343 (7)	
H16	0.9148	0.4621	−0.0243	0.041*	
C5	0.6704 (2)	0.3861 (2)	0.17153 (12)	0.0158 (5)	
C17	0.7302 (3)	0.3874 (3)	−0.05183 (14)	0.0335 (7)	
H17	0.7454	0.3890	−0.0994	0.040*	
C9	0.8593 (3)	0.3661 (2)	0.26428 (13)	0.0213 (6)	
H9	0.8245	0.4367	0.2848	0.026*	
C7	0.5206 (3)	0.3499 (3)	0.28064 (12)	0.0237 (6)	
H7A	0.5368	0.4344	0.2915	0.036*	
H7B	0.5902	0.3004	0.3030	0.036*	
H7C	0.4291	0.3265	0.2966	0.036*	
C2	0.1796 (3)	0.4270 (3)	0.14409 (15)	0.0298 (7)	
H2A	0.1033	0.4348	0.1770	0.036*	
H2B	0.1418	0.4018	0.0997	0.036*	
C4	0.5295 (3)	0.3314 (2)	0.20112 (12)	0.0169 (5)	
C18	0.6074 (3)	0.3418 (3)	−0.02570 (13)	0.0304 (7)	
H18	0.5394	0.3120	−0.0558	0.037*	
C1	0.2605 (3)	0.5484 (3)	0.13726 (13)	0.0205 (6)	
H1	0.2242	0.6061	0.1718	0.025*	
C20	0.2515 (3)	0.6070 (3)	0.06651 (14)	0.0282 (7)	
C6	0.5148 (3)	0.1927 (2)	0.18810 (13)	0.0213 (6)	
H6A	0.4251	0.1654	0.2046	0.032*	
H6B	0.5877	0.1500	0.2123	0.032*	
H6C	0.5223	0.1766	0.1393	0.032*	
C21A	0.2705 (6)	0.5271 (5)	0.0051 (2)	0.0364 (14)	0.600 (4)
H21A	0.2875	0.4440	0.0107	0.044*	0.600 (4)
C22A	0.2626 (6)	0.5774 (5)	−0.0610 (2)	0.0391 (14)	0.600 (4)
H22A	0.2752	0.5273	−0.0994	0.047*	0.600 (4)
C23A	0.2373 (7)	0.6970 (6)	−0.0704 (3)	0.0343 (15)	0.600 (4)
H23A	0.2331	0.7297	−0.1149	0.041*	0.600 (4)
C24A	0.2175 (9)	0.7703 (5)	−0.0134 (3)	0.0521 (19)	0.600 (4)
H24A	0.1997	0.8532	−0.0192	0.062*	0.600 (4)
C25A	0.2240 (7)	0.7205 (5)	0.0536 (3)	0.0458 (16)	0.600 (4)
H25A	0.2077	0.7722	0.0910	0.055*	0.600 (4)
C21B	0.3699 (8)	0.6581 (8)	0.0362 (4)	0.036 (2)	0.400 (4)
H21B	0.4575	0.6488	0.0567	0.044*	0.400 (4)
C22B	0.3554 (9)	0.7233 (8)	−0.0252 (4)	0.042 (2)	0.400 (4)
H22B	0.4352	0.7480	−0.0490	0.050*	0.400 (4)
C23B	0.2253 (11)	0.7519 (10)	−0.0514 (5)	0.039 (3)	0.400 (4)
H23B	0.2167	0.7919	−0.0937	0.046*	0.400 (4)
C24B	0.1101 (9)	0.7207 (8)	−0.0144 (4)	0.036 (2)	0.400 (4)
H24B	0.0223	0.7444	−0.0304	0.043*	0.400 (4)
C25B	0.1192 (8)	0.6542 (7)	0.0469 (4)	0.0310 (18)	0.400 (4)
H25B	0.0404	0.6412	0.0743	0.037*	0.400 (4)

### Atomic displacement parameters (Å^2^)

**Table:**

	*U*^11^	*U*^22^	*U*^33^	*U*^12^	*U*^13^	*U*^23^
O1	0.0155 (8)	0.0262 (10)	0.0375 (11)	−0.0019 (9)	−0.0028 (8)	0.0104 (9)
O2	0.0163 (8)	0.0146 (8)	0.0239 (9)	−0.0003 (8)	−0.0017 (8)	−0.0015 (7)
C14	0.0205 (12)	0.0155 (12)	0.0190 (12)	0.0054 (12)	−0.0007 (11)	0.0015 (10)
C3	0.0157 (11)	0.0219 (13)	0.0152 (11)	−0.0014 (12)	0.0009 (10)	0.0008 (10)
C15	0.0225 (12)	0.0324 (15)	0.0219 (13)	0.0034 (14)	−0.0003 (11)	0.0053 (12)
N6	0.0182 (10)	0.0192 (11)	0.0215 (11)	0.0021 (10)	−0.0019 (9)	0.0015 (9)
C13	0.0220 (12)	0.0245 (14)	0.0176 (12)	0.0033 (12)	0.0024 (11)	0.0019 (11)
C8	0.0173 (11)	0.0181 (12)	0.0158 (11)	−0.0014 (11)	0.0004 (10)	0.0024 (10)
C11	0.0166 (11)	0.0316 (16)	0.0315 (14)	−0.0022 (14)	−0.0026 (12)	0.0128 (13)
C12	0.0235 (12)	0.0234 (13)	0.0249 (13)	0.0060 (13)	0.0031 (11)	0.0046 (12)
C10	0.0247 (12)	0.0291 (15)	0.0270 (13)	−0.0044 (14)	−0.0074 (12)	0.0032 (12)
C19	0.0263 (13)	0.0235 (14)	0.0197 (12)	0.0000 (13)	−0.0006 (11)	−0.0004 (11)
C16	0.0277 (14)	0.0450 (19)	0.0302 (15)	0.0087 (16)	0.0110 (13)	0.0124 (14)
C5	0.0165 (11)	0.0128 (11)	0.0179 (11)	−0.0007 (11)	−0.0013 (10)	−0.0006 (10)
C17	0.0447 (17)	0.0420 (18)	0.0138 (13)	0.0139 (16)	0.0081 (13)	0.0005 (12)
C9	0.0212 (12)	0.0198 (13)	0.0228 (13)	−0.0023 (11)	−0.0019 (11)	−0.0003 (11)
C7	0.0245 (12)	0.0286 (15)	0.0181 (12)	0.0015 (14)	0.0019 (11)	0.0044 (11)
C2	0.0168 (12)	0.0322 (16)	0.0403 (16)	0.0020 (14)	−0.0066 (12)	0.0118 (14)
C4	0.0170 (11)	0.0153 (12)	0.0184 (12)	−0.0004 (12)	0.0000 (10)	0.0009 (11)
C18	0.0395 (15)	0.0332 (16)	0.0187 (13)	0.0070 (16)	−0.0034 (12)	−0.0055 (12)
C1	0.0164 (11)	0.0239 (14)	0.0212 (13)	0.0029 (12)	0.0030 (10)	0.0025 (11)
C20	0.0180 (12)	0.0408 (18)	0.0257 (14)	0.0023 (14)	−0.0024 (11)	0.0094 (13)
C6	0.0211 (12)	0.0183 (13)	0.0244 (13)	−0.0014 (12)	−0.0031 (11)	0.0011 (11)
C21A	0.055 (3)	0.032 (3)	0.022 (2)	0.016 (3)	−0.005 (2)	−0.005 (2)
C22A	0.053 (3)	0.043 (3)	0.021 (2)	0.010 (3)	−0.002 (2)	−0.003 (2)
C23A	0.040 (3)	0.046 (4)	0.017 (3)	−0.003 (3)	−0.006 (2)	0.005 (3)
C24A	0.098 (5)	0.024 (3)	0.035 (3)	−0.004 (4)	−0.008 (4)	0.009 (3)
C25A	0.085 (4)	0.029 (3)	0.023 (2)	−0.002 (3)	−0.005 (3)	−0.002 (2)
C21B	0.034 (4)	0.045 (4)	0.029 (4)	−0.004 (4)	0.001 (3)	0.011 (4)
C22B	0.049 (4)	0.048 (5)	0.029 (4)	−0.013 (4)	−0.001 (4)	0.016 (4)
C23B	0.062 (5)	0.041 (6)	0.013 (4)	0.004 (5)	0.007 (4)	0.007 (4)
C24B	0.039 (4)	0.037 (4)	0.033 (4)	0.010 (4)	−0.006 (3)	0.017 (3)
C25B	0.031 (3)	0.036 (4)	0.027 (3)	−0.003 (4)	−0.006 (3)	0.001 (3)

### Geometric parameters (Å, º)

**Table:**

O1—C3	1.355 (3)	C7—H7C	0.9600
O1—C2	1.453 (3)	C2—C1	1.542 (4)
O2—C5	1.442 (3)	C2—H2A	0.9700
O2—H2	0.86 (3)	C2—H2B	0.9700
C14—C19	1.383 (4)	C4—C6	1.545 (3)
C14—C15	1.396 (4)	C18—H18	0.9300
C14—C5	1.548 (3)	C1—C20	1.510 (4)
C3—N6	1.274 (3)	C1—H1	0.9800
C3—C4	1.513 (3)	C20—C25A	1.293 (6)
C15—C16	1.382 (4)	C20—C21B	1.390 (8)
C15—H15	0.9300	C20—C25B	1.415 (7)
N6—C1	1.483 (3)	C20—C21A	1.483 (5)
C13—C12	1.388 (4)	C6—H6A	0.9600
C13—C8	1.391 (4)	C6—H6B	0.9600
C13—H13	0.9300	C6—H6C	0.9600
C8—C9	1.395 (3)	C21A—C22A	1.391 (6)
C8—C5	1.531 (3)	C21A—H21A	0.9300
C11—C12	1.379 (4)	C22A—C23A	1.343 (8)
C11—C10	1.387 (4)	C22A—H22A	0.9300
C11—H11	0.9300	C23A—C24A	1.375 (8)
C12—H12	0.9300	C23A—H23A	0.9300
C10—C9	1.389 (4)	C24A—C25A	1.403 (7)
C10—H10	0.9300	C24A—H24A	0.9300
C19—C18	1.406 (4)	C25A—H25A	0.9300
C19—H19	0.9300	C21B—C22B	1.389 (9)
C16—C17	1.380 (4)	C21B—H21B	0.9300
C16—H16	0.9300	C22B—C23B	1.377 (12)
C5—C4	1.578 (3)	C22B—H22B	0.9300
C17—C18	1.370 (4)	C23B—C24B	1.355 (11)
C17—H17	0.9300	C23B—H23B	0.9300
C9—H9	0.9300	C24B—C25B	1.391 (9)
C7—C4	1.549 (3)	C24B—H24B	0.9300
C7—H7A	0.9600	C25B—H25B	0.9300
C7—H7B	0.9600		
			
C3—O1—C2	106.09 (19)	C6—C4—C7	106.5 (2)
C5—O2—H2	98 (2)	C3—C4—C5	109.78 (19)
C19—C14—C15	118.0 (2)	C6—C4—C5	113.0 (2)
C19—C14—C5	125.6 (2)	C7—C4—C5	110.8 (2)
C15—C14—C5	116.3 (2)	C17—C18—C19	120.8 (3)
N6—C3—O1	118.2 (2)	C17—C18—H18	119.6
N6—C3—C4	125.7 (2)	C19—C18—H18	119.6
O1—C3—C4	115.9 (2)	N6—C1—C20	112.5 (2)
C16—C15—C14	121.5 (3)	N6—C1—C2	103.5 (2)
C16—C15—H15	119.3	C20—C1—C2	114.5 (2)
C14—C15—H15	119.3	N6—C1—H1	108.7
C3—N6—C1	107.5 (2)	C20—C1—H1	108.7
C12—C13—C8	121.0 (2)	C2—C1—H1	108.7
C12—C13—H13	119.5	C25A—C20—C21B	72.4 (5)
C8—C13—H13	119.5	C25A—C20—C25B	54.4 (4)
C13—C8—C9	117.8 (2)	C21B—C20—C25B	117.8 (4)
C13—C8—C5	121.1 (2)	C25A—C20—C21A	115.9 (4)
C9—C8—C5	121.1 (2)	C21B—C20—C21A	78.6 (4)
C12—C11—C10	118.9 (2)	C25B—C20—C21A	96.3 (4)
C12—C11—H11	120.6	C25A—C20—C1	126.4 (3)
C10—C11—H11	120.6	C21B—C20—C1	120.3 (4)
C11—C12—C13	120.8 (3)	C25B—C20—C1	116.6 (4)
C11—C12—H12	119.6	C21A—C20—C1	117.7 (3)
C13—C12—H12	119.6	C4—C6—H6A	109.5
C11—C10—C9	120.6 (3)	C4—C6—H6B	109.5
C11—C10—H10	119.7	H6A—C6—H6B	109.5
C9—C10—H10	119.7	C4—C6—H6C	109.5
C14—C19—C18	120.2 (3)	H6A—C6—H6C	109.5
C14—C19—H19	119.9	H6B—C6—H6C	109.5
C18—C19—H19	119.9	C22A—C21A—C20	119.5 (4)
C17—C16—C15	120.0 (3)	C22A—C21A—H21A	120.2
C17—C16—H16	120.0	C20—C21A—H21A	120.2
C15—C16—H16	120.0	C23A—C22A—C21A	121.3 (5)
O2—C5—C8	106.15 (19)	C23A—C22A—H22A	119.4
O2—C5—C14	107.39 (19)	C21A—C22A—H22A	119.4
C8—C5—C14	109.5 (2)	C22A—C23A—C24A	119.0 (5)
O2—C5—C4	108.78 (19)	C22A—C23A—H23A	120.5
C8—C5—C4	109.78 (18)	C24A—C23A—H23A	120.5
C14—C5—C4	114.86 (19)	C23A—C24A—C25A	120.3 (5)
C18—C17—C16	119.5 (2)	C23A—C24A—H24A	119.9
C18—C17—H17	120.3	C25A—C24A—H24A	119.9
C16—C17—H17	120.3	C20—C25A—C24A	124.0 (5)
C10—C9—C8	120.9 (2)	C20—C25A—H25A	118.0
C10—C9—H9	119.5	C24A—C25A—H25A	118.0
C8—C9—H9	119.5	C22B—C21B—C20	118.9 (6)
C4—C7—H7A	109.5	C22B—C21B—H21B	120.5
C4—C7—H7B	109.5	C20—C21B—H21B	120.5
H7A—C7—H7B	109.5	C23B—C22B—C21B	121.4 (7)
C4—C7—H7C	109.5	C23B—C22B—H22B	119.3
H7A—C7—H7C	109.5	C21B—C22B—H22B	119.3
H7B—C7—H7C	109.5	C24B—C23B—C22B	118.7 (8)
O1—C2—C1	104.63 (19)	C24B—C23B—H23B	120.7
O1—C2—H2A	110.8	C22B—C23B—H23B	120.7
C1—C2—H2A	110.8	C23B—C24B—C25B	122.0 (8)
O1—C2—H2B	110.8	C23B—C24B—H24B	119.0
C1—C2—H2B	110.8	C25B—C24B—H24B	119.0
H2A—C2—H2B	108.9	C24B—C25B—C20	118.3 (6)
C3—C4—C6	111.8 (2)	C24B—C25B—H25B	120.9
C3—C4—C7	104.6 (2)	C20—C25B—H25B	120.9
			
C2—O1—C3—N6	1.0 (3)	C14—C5—C4—C6	65.4 (3)
C2—O1—C3—C4	176.6 (2)	O2—C5—C4—C7	−54.8 (3)
C19—C14—C15—C16	−0.6 (4)	C8—C5—C4—C7	61.0 (3)
C5—C14—C15—C16	−179.0 (3)	C14—C5—C4—C7	−175.2 (2)
O1—C3—N6—C1	0.6 (3)	C16—C17—C18—C19	0.3 (5)
C4—C3—N6—C1	−174.6 (2)	C14—C19—C18—C17	−0.8 (4)
C12—C13—C8—C9	0.1 (4)	C3—N6—C1—C20	−125.9 (2)
C12—C13—C8—C5	179.9 (2)	C3—N6—C1—C2	−1.8 (3)
C10—C11—C12—C13	0.9 (4)	O1—C2—C1—N6	2.3 (3)
C8—C13—C12—C11	−0.8 (4)	O1—C2—C1—C20	125.2 (2)
C12—C11—C10—C9	−0.4 (4)	N6—C1—C20—C25A	−109.5 (5)
C15—C14—C19—C18	0.9 (4)	C2—C1—C20—C25A	132.7 (5)
C5—C14—C19—C18	179.2 (3)	N6—C1—C20—C21B	−19.8 (5)
C14—C15—C16—C17	0.1 (5)	C2—C1—C20—C21B	−137.6 (5)
C13—C8—C5—O2	−154.6 (2)	N6—C1—C20—C25B	−173.3 (4)
C9—C8—C5—O2	25.2 (3)	C2—C1—C20—C25B	68.9 (5)
C13—C8—C5—C14	−38.9 (3)	N6—C1—C20—C21A	72.9 (4)
C9—C8—C5—C14	140.8 (2)	C2—C1—C20—C21A	−44.9 (4)
C13—C8—C5—C4	88.0 (3)	C25A—C20—C21A—C22A	2.0 (7)
C9—C8—C5—C4	−92.2 (3)	C21B—C20—C21A—C22A	−61.8 (6)
C19—C14—C5—O2	−118.7 (3)	C25B—C20—C21A—C22A	55.3 (6)
C15—C14—C5—O2	59.6 (3)	C1—C20—C21A—C22A	179.9 (4)
C19—C14—C5—C8	126.5 (3)	C20—C21A—C22A—C23A	−0.4 (9)
C15—C14—C5—C8	−55.2 (3)	C21A—C22A—C23A—C24A	−0.6 (10)
C19—C14—C5—C4	2.4 (4)	C22A—C23A—C24A—C25A	0.1 (12)
C15—C14—C5—C4	−179.3 (2)	C21B—C20—C25A—C24A	64.7 (8)
C15—C16—C17—C18	0.1 (5)	C25B—C20—C25A—C24A	−81.3 (8)
C11—C10—C9—C8	−0.3 (4)	C21A—C20—C25A—C24A	−2.6 (9)
C13—C8—C9—C10	0.4 (4)	C1—C20—C25A—C24A	179.8 (6)
C5—C8—C9—C10	−179.4 (2)	C23A—C24A—C25A—C20	1.7 (12)
C3—O1—C2—C1	−2.1 (3)	C25A—C20—C21B—C22B	−50.5 (7)
N6—C3—C4—C6	−157.5 (2)	C25B—C20—C21B—C22B	−19.6 (10)
O1—C3—C4—C6	27.3 (3)	C21A—C20—C21B—C22B	71.6 (7)
N6—C3—C4—C7	87.7 (3)	C1—C20—C21B—C22B	−172.8 (6)
O1—C3—C4—C7	−87.5 (3)	C20—C21B—C22B—C23B	8.8 (14)
N6—C3—C4—C5	−31.2 (3)	C21B—C22B—C23B—C24B	3.3 (16)
O1—C3—C4—C5	153.5 (2)	C22B—C23B—C24B—C25B	−4.1 (16)
O2—C5—C4—C3	60.2 (2)	C23B—C24B—C25B—C20	−7.0 (13)
C8—C5—C4—C3	176.0 (2)	C25A—C20—C25B—C24B	55.8 (7)
C14—C5—C4—C3	−60.2 (3)	C21B—C20—C25B—C24B	18.8 (9)
O2—C5—C4—C6	−174.25 (19)	C21A—C20—C25B—C24B	−61.7 (7)
C8—C5—C4—C6	−58.5 (2)	C1—C20—C25B—C24B	173.0 (6)

### Hydrogen-bond geometry (Å, º)

Cg1, Cg2, Cg3 and Cg4 are the centroids of the C14–C19, C8–C13, C14–C19 and C20–C25 rings, respectively.

**Table:**

*D*—H···*A*	*D*—H	H···*A*	*D*···*A*	*D*—H···*A*
O2—H2···N6	0.86 (3)	1.89 (3)	2.712 (3)	159 (3)
C1—H1···*Cg*2^i^	0.98	2.80	3.771 (3)	173
C2—H2*A*···*Cg*2^ii^	0.97	2.86	3.589 (3)	132
C21*A*—H21*A*···*Cg*1	0.93	2.81	3.058 (4)	97
C24*B*—H24*B*···*Cg*3^iii^	0.93	2.87	3.776 (6)	166
C24*B*—H24*B*···*Cg*4^iii^	0.93	2.97	3.785 (9)	147

Symmetry codes: (i) −*x*+1, *y*+1/2, −*z*+1/2; (ii) *x*−1, *y*, *z*; (iii) *x*−1/2, −*y*+3/2, −*z*.
